# Primary Cutaneous Gamma-Delta T-cell Lymphoma: A Case Report and Review of Literature

**DOI:** 10.7759/cureus.35442

**Published:** 2023-02-25

**Authors:** Yashvin Onkarappa Mangala, Ifeanyichukwu D Onukogu, Catherine M Breen, Gerald A Colvin

**Affiliations:** 1 Hematology and Medical Oncology, Roger Williams Medical Center, Providence, USA; 2 Pathology, Roger Williams Medical Center, Providence, USA

**Keywords:** cutaneous t-cell lymphoma, t cell, lymphoma, t-cell lymphoma, gamma-delta t-cell

## Abstract

Primary cutaneous gamma-delta T-cell lymphoma (PCGD-TCL) is a rare type of lymphoma, representing less than 1% of all cutaneous T-cell lymphomas. It is typically aggressive and chemotherapy-refractory. Hence, most institutions tend to employ intensive chemotherapy followed by stem cell transplantation although there is no standard of care established. We report a case of PCGD-TCL and discuss the challenges associated with the diagnosis and management of PCGD-TCL.

## Introduction

Primary cutaneous gamma-delta T-cell lymphoma (PCGD-TCL) is a rare and highly aggressive subtype of cutaneous T-cell lymphomas (CTCLs) characterized by clonal proliferation of mature, activated gamma-delta T-cells with a cytotoxic phenotype [1]. The first case descriptions of PCGD-TCL were made in 1991, a case of disseminated pagetoid reticulosis with epidermotrophic gamma-delta infiltration by Berti et. al [2], and T-cell receptor (TCR) delta-positive subcutaneous plaques without epidermotrophism by Burg et al. [3]. The World Health Organization-European Organization for Research and Treatment of Cancer (WHO-EORTC) classification for cutaneous lymphomas included PCGD-TCL as a provisional entity in 2005 and later as a definitive entity in 2008 [1,4]. Only a small number of cases have been reported given the rarity of this subtype.

In this article, we describe a case of PCGD-TCL seen in our institution and review the existing literature on PCGD-TCL.

## Case presentation

**Figure 1:**
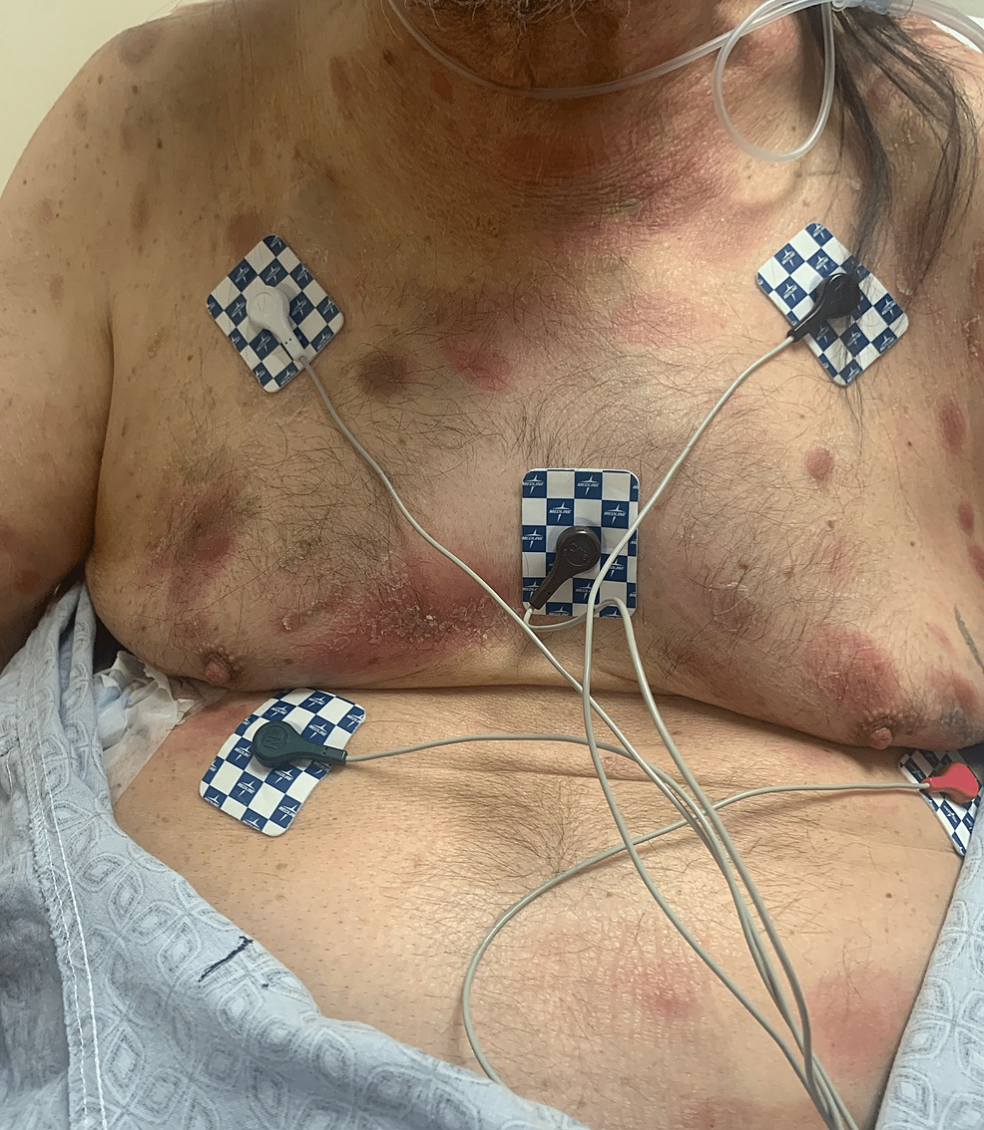
Photograph showing cutaneous lesions over the patient's chest and abdomen

**Figure 2:**
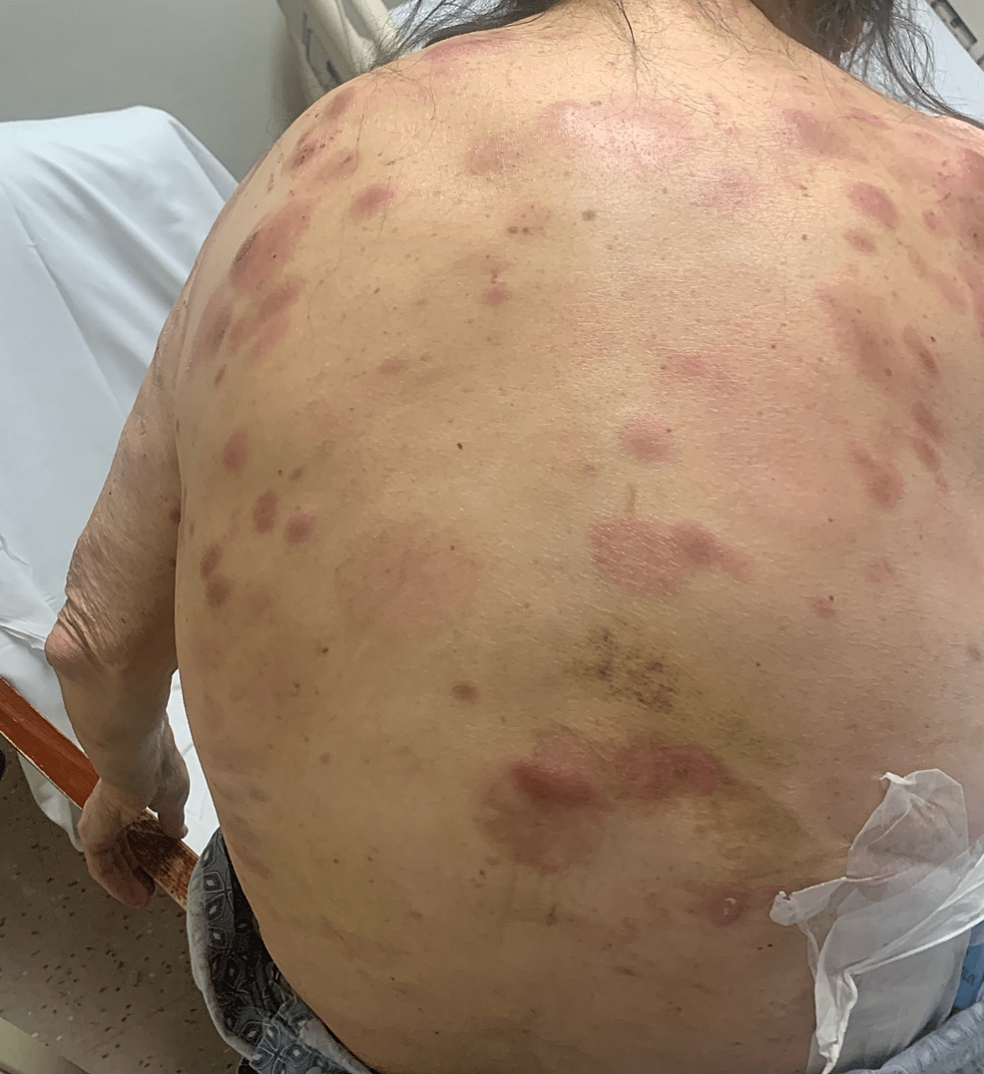
Photograph showing diffuse cutaneous lesions over the posterior aspect of the trunk

A 68-year-old gentleman presented to our clinic with diffuse skin lesions after being referred by a dermatologist. The patient initially noticed two red, itchy spots over his right lower extremity a year prior to his presentation to the clinic which disappeared shortly but then he noticed a similar rash on his left thigh. Due to health insurance issues, he could not undergo dermatological evaluation for almost a year. In the meanwhile, he developed more skin lesions throughout his body, including the face, chest, abdomen, and upper extremities (Figure 1 and Figure 2).

**Figure 3:**
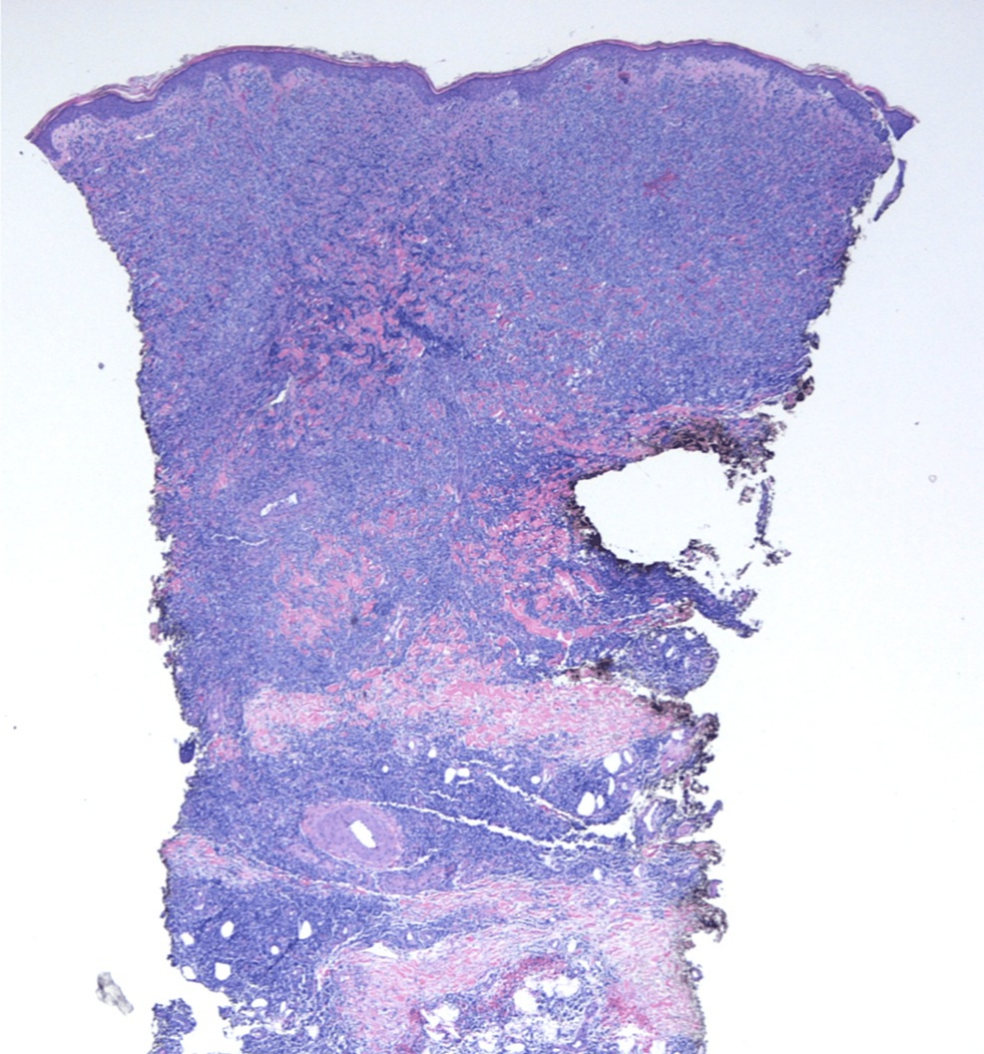
Low-power microscopic view with 20x magnification showing a dense infiltrate present diffusely in the dermis

**Figure 4:**
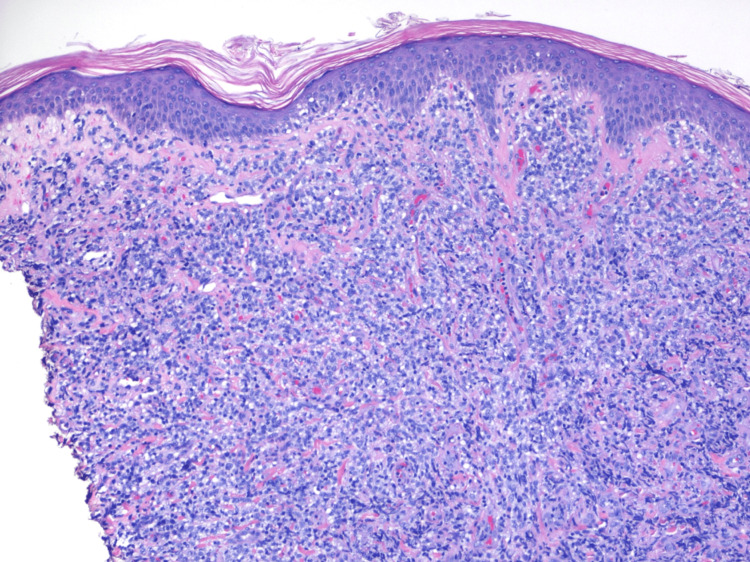
High-power microscopic view with 100x magnification showing dermal infiltrate of medium-sized lymphocytes with background small lymphocytes and minimal epidermal involvement

He had no constitutional symptoms such as fever, drenching night sweats, or weight loss. A punch biopsy of a skin lesion over his left thigh was done by his dermatologist which revealed dense, diffuse dermal and sub-cutaneous infiltrate composed of medium-sized cells with admixed small lymphocytes, no lymphoid follicles were identified (Figure 3 and Figure 4).

**Figure 5:**
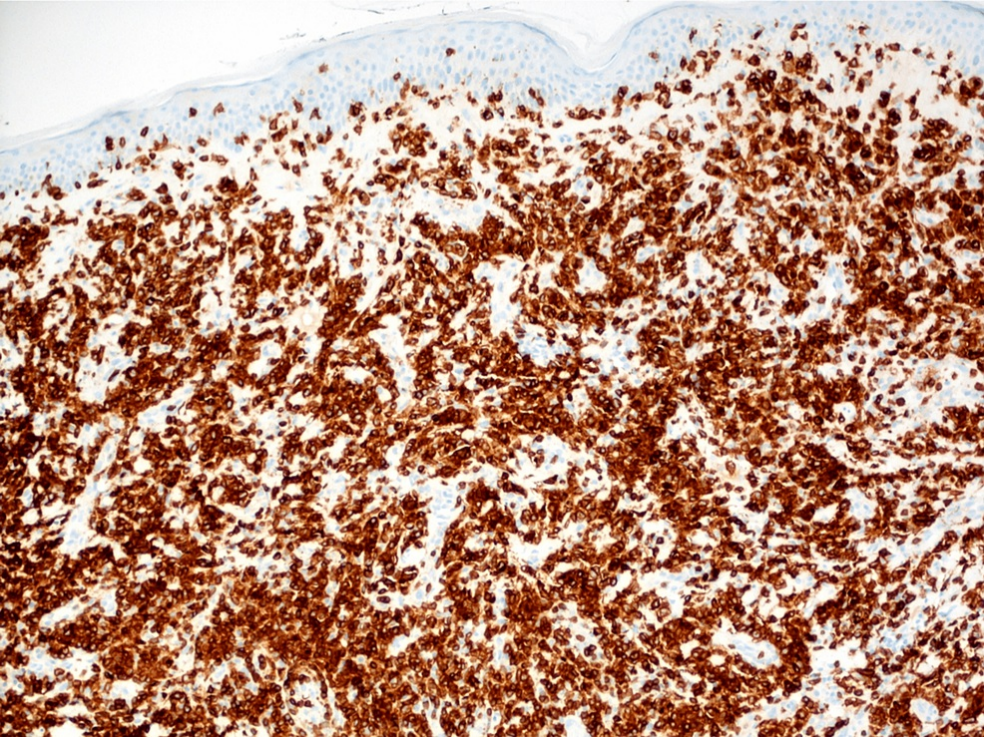
CD3 immunohistochemical stain highlighting T-lymphocytes in 100x magnification

**Figure 6:**
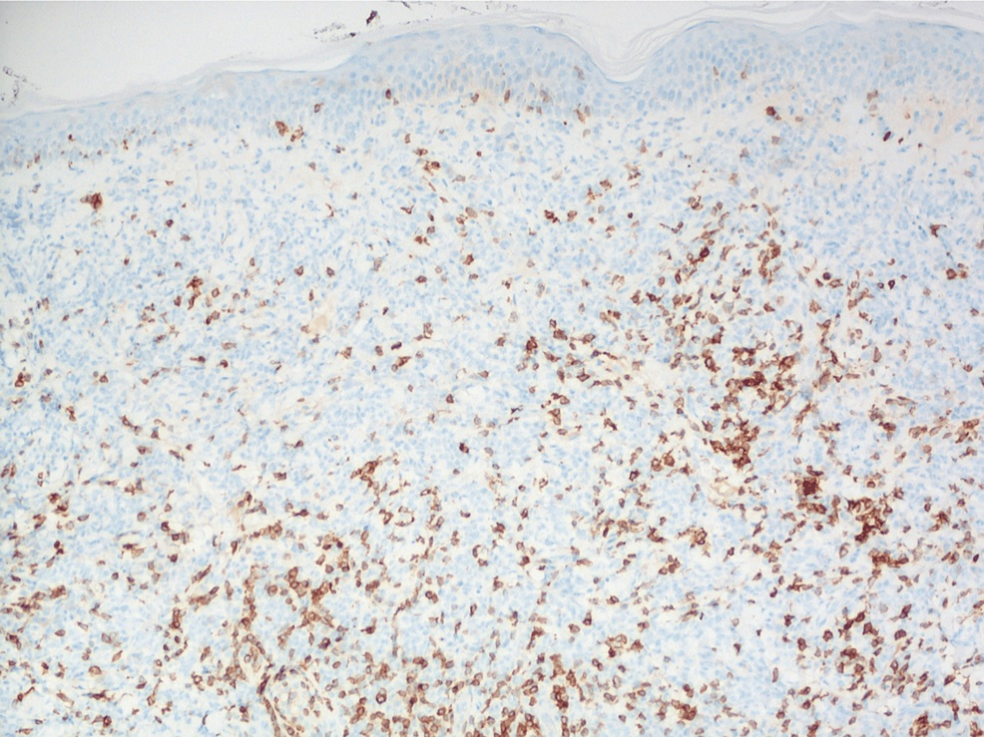
CD5 immunohistochemical staining with the loss of expression in many of the CD3-positive lymphocytes on 100x magnification

Immunohistochemical (IHC) analysis showed a strongly positive CD3 stain highlighting T-lymphocytes, with the loss of expression of CD5 (Figure 5 and Figure 6).

**Figure 7:**
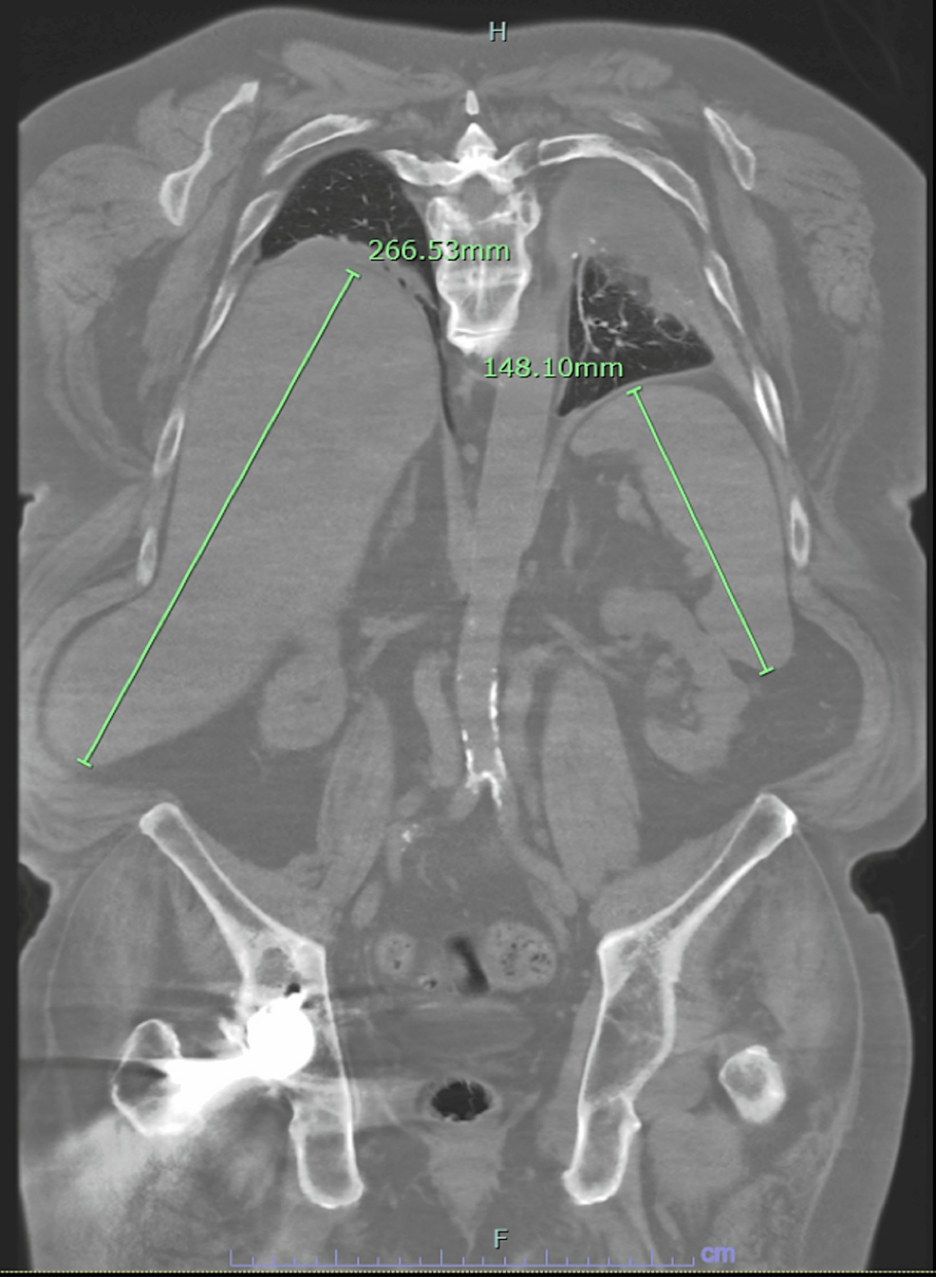
CT imaging showing hepatosplenomegaly CT: computed tomography

IHC also showed the expression of CD2, TCR-gamma/delta, a subset positive for CD56 but no expression of CD30, CD123, TDT, and Beta-F1. Overall, the pathological findings were consistent with a diagnosis of PCGD-TCL. Computed tomography (CT) imaging of the chest, abdomen, and pelvis performed later showed moderate splenomegaly (14.8 cm) and hepatomegaly (27 cm) as shown in Figure 7.

The patient’s case was discussed in a multidisciplinary tumor board meeting given the rarity of the disease and no randomized trials being available to guide the treatment. He was started on cyclophosphamide, vincristine, doxorubicin, and prednisone (CHOP) regimen. After the first cycle of CHOP, he developed a large de-epithelialized lesion with areas of full-thickness necrosis, measuring approximately 30 x 20 cm over the right flank region. He underwent surgical debridement and received antibiotic therapy followed by wound care. It was decided to hold off on any further chemotherapy due to ongoing morbidity. Over the period of the next few months, the patient’s clinical status continued to deteriorate with recurrent wound infections and encephalopathy with repeated hospitalizations requiring intravenous antibiotic therapy and discharge to a rehabilitation facility. The patient contracted COVID-19 at his skilled nursing facility and had to be re-hospitalized again. He had a complicated hospital course with a fall leading to cardiac arrest requiring cardiopulmonary resuscitation and transfer to Intensive Care Unit. CT imaging at that time revealed severe spinal cord injury at the level of the thoracolumbar junction with extensive prevertebral hemorrhage, critical narrowing of the aorta, hypo-perfusion of the right kidney, acute vascular injury involving the abdominal aorta, along with multifocal chest wall trauma involving the sternum and multiple bilateral ribs. A family discussion was held and the patient’s code status was changed to comfort measures only given the non-life-sustainable nature of his injuries.

## Discussion

PCGD-TCL represents <1% of all CTCLs [2]. These tumors originate from gamma-delta T-cell lymphocytes which naturally play a role in the innate, non-specific immune response. Gamma-delta T-cells arise from thymic precursors in bone marrow with no major histocompatibility complex restriction. Chronic immunosuppression and prolonged antigenic exposure are believed to play major roles in lymphomagenesis.

Most affected patients are adults, with a median age of onset of around 60 years [5]. There is no gender preference and both sexes are equally affected [6]. The patients usually present with disseminated skin lesions mostly on the extremities, which can be seen in the form of rapidly progressing plaques, and ulcero-necrotic nodules. There is frequent involvement of mucosal and other extranodal sites except for the involvement of lymph nodes, spleen, or bone marrow which is uncommon [1]. Constitutional symptoms such as fevers, night sweats, and weight loss are not uncommon. Daniels et al. [7] identified two molecular subtypes of PCGD-TCL; Vdelta-1 subtype with the epidermis and dermis involvement and Vdelta-2 with the panniculitis form. PCGD-TCL can be associated with hemophagocytic lymphohistiocytosis in patients with panniculitis-like tumors [8]. Microscopically, skin infiltration can follow three different patterns: epidermotropic, dermal, and subcutaneous, even within the same patient and/or the same lesion [9]. Angiocentricity and tissue necrosis are seen in histology. The cells are usually medium to large in size with clumped chromatin and are characteristically positive for TCR gamma/delta, CD3, CD2, and CD56, and negative for beta-F1, CD5, CD4, and CD8 [10].

Due to its rarity, there are no clinical trials addressing the treatment of PCGD-TCL. The treatment guidelines are mostly based on case reports. The most commonly used regimen remains to be an anthracycline-based regimen similar to CHOP. The response is seen in about 50% of the patients, but the majority of them relapse with tumor progression and death. The other treatments used include methotrexate and narrow-band ultraviolet radiation in cases presenting with a patch/plaque [11]. Bexarotene has been used as a single agent or in combination with a reasonably good response [12]. Some have tried autologous and allogeneic hematopoeitic stem cell transplants as well.

The overall prognosis is extremely poor due to the aggressive and chemo-resistant nature of PCGD-TCL. The median survival is about 15 months with five-year overall survival is about 10% [10]. The indolent subtypes may have a slightly better prognosis [13]. The poor prognostic factors include age >40 years, subcutaneous involvement and ulceration, CD56, CD95 expression without CD8, central nervous system involvement, and hemophagocytic lymphohistiocytosis syndrome [14,15].

## Conclusions

To date, due to its rarity, there are not many studies done on PCGD-TCL, a type of CTCL. Most published articles are case reports. The most commonly used regimen is doxorubicin-based therapies such as CHOP. Others include Bexarotene and stem cell transplantation. The overall prognosis is poor with an estimated 50% response to treatment which often relapses with disease progression leading to death. Further clinical studies are needed to improve the efficacy of clinical treatment and achieve better outcomes in patients with PCGD-TCL.
